# Rare-Disease Diagnosis on the ZebraMap Multimodal Case Report Dataset: A Hybrid Pipeline with Grounded Explainability

**DOI:** 10.3390/s26113582

**Published:** 2026-06-04

**Authors:** Md Sanzidul Islam, Amani Jamal, Ali Alkhathlan

**Affiliations:** 1 Department of Computer Science, Faculty of Computing and Information Technology, King Abdulaziz University, Jeddah 21589, Saudi Arabia; atjamal@kau.edu.sa (A.J.); analkhathlan@kau.edu.sa (A.A.); 2 Department of Computer Science, Faculty of Science and Information Technology, Daffodil International University, Birulia 1216, Bangladesh; 3 Center of Research Excellence in Artificial Intelligence and Data Science, King Abdulaziz University, Jeddah 21589, Saudi Arabia

**Keywords:** rare-disease diagnosis, clinical decision support, benchmarking, explainability, large language models, caption-mediated multimodal fusion, information retrieval

## Abstract

Rare-disease diagnosis is difficult because clinicians must identify plausible conditions from a large, severely imbalanced disease space using evidence distributed across clinical narratives, structured findings, and image-linked descriptions. This paper presents a hybrid pipeline with caption-mediated multimodal fusion for ranked rare-disease diagnosis and grounded explanation, developed and evaluated on the ZebraMap multimodal case-report dataset (69,146 structured cases; 1727 diseases). Grouped train–validation–test splitting by source article was applied to prevent leakage, and a sequential pipeline was constructed combining BM25 lexical retrieval, a class-balanced TF–IDF classifier, MedCPT dense retrieval and cross-encoder reranking, caption-based image-aware late fusion, and post hoc grounded explanation generation. The final pipeline achieved test MRR 0.3905 and Recall@10 0.5507 (nDCG@10 0.4273), while the strongest individual component, the class-balanced TF–IDF classifier, reached MRR 0.4200 and Recall@10 0.6279; the hybrid pipeline therefore integrates ranking with grounded explanation rather than maximizing single-metric diagnostic accuracy. On 256 explained cases, the explanation module achieved citation coverage 0.7334 and usefulness 3.8734, exposing a tradeoff between diagnostic accuracy and explanation richness. These results indicate that a hybrid retrieval-and-classification approach can support ranked rare-disease differential diagnosis and that grounded explanation quality can be evaluated quantitatively, extending computational support for the prolonged rare-disease diagnostic process.

## 1. Introduction

Rare-disease diagnosis remains a significant clinical problem. Although 3.5–5.9% of the global population is affected by one of more than 6000 rare diseases [1,2], each condition is encountered infrequently by individual clinicians, creating a setting in which diagnosis is often delayed and dependent on repeated specialist referrals [3,4,5]. Computational methods are relevant here not as replacements for clinical judgment but as tools that can organize large disease spaces, prioritize plausible candidates, and attach structured evidence to support specialist review at earlier stages of care.

From a computer-science perspective, rare-disease diagnosis is not merely another multiclass classification problem. The label space is large, the class distribution is severely imbalanced, and the evidence associated with each case is heterogeneous. Clinical narratives may contain long descriptive passages, structured symptom inventories may only partially capture the phenotype, diagnostic-method fields encode procedural context, and images or image-linked captions may add auxiliary information that is neither uniformly available nor trivially fused with text. These properties make flat one-shot prediction an inadequate formulation for the task. A more realistic alternative is ranked differential diagnosis, in which a system returns an ordered list of candidate diseases and is evaluated on whether the true diagnosis appears near the top of that list. This paper operationalizes that idea as full-label-space top-*k* ranking, where a prediction is counted as successful if the correct disease appears among the first *k* returned candidates. This formulation is consistent with standard ranked-retrieval evaluation practice [6] and with clinical decision-support settings in which ranked candidate lists guide downstream review [7].

Recent work in rare-disease AI has advanced along several distinct lines, though the field remains fragmented in methodology. One line of studies has focused on decision-support systems and machine learning models for rare diseases, documenting recurring weaknesses in study design such as heterogeneous cohorts, inconsistent validation procedures, limited external testing, and narrow disease coverage [8,9,10]. A second line has shifted attention toward large language models and retrieval-augmented diagnostic support: RareDxGPT demonstrated that retrieval augmentation could improve ChatGPT 3.5-based diagnosis on a small phenotype-oriented case set [11], while RareArena scaled rare-disease language-model evaluation to a much larger corpus derived from PubMed Central case reports [12]. A third line, situated in broader clinical AI, has shown that multimodal integration and explanation generation are promising but difficult to validate rigorously [13,14,15]. Collectively, these strands show progress in model capability, dataset construction, and prompting-based evaluation. However, they also reveal that benchmark design, modality integration, and explanation assessment are typically addressed in isolation rather than within a unified downstream diagnosis framework.

This fragmentation exposes the central gap addressed here. To the best of the authors’ knowledge, no existing rare-disease study provides a downstream benchmark built on a published multimodal case-report resource that simultaneously supports full-label-space disease ranking, strong lexical and supervised baselines, dense retrieval and reranking, multimodal late fusion, and quantitative evaluation of grounded explanation. Text-only prompting benchmarks address language-model performance, but they do not establish how retrieval, classification, reranking, and multimodal evidence should be combined in a reproducible disease-ranking pipeline. Conversely, data-resource papers provide the substrate for experimentation but do not define a benchmark task, split protocol, or evaluation framework for diagnostic reasoning. ZebraMap was introduced as a multimodal rare-disease knowledge map built from case reports, disease summaries, structured clinical fields, and linked images [16]. What remains unresolved is whether that resource can function as a rigorous benchmark for rare-disease diagnosis rather than only as a curated dataset, and whether grounded explanation can be evaluated on top of fixed diagnosis outputs in a reproducible and analytically useful manner.

Building on this gap, the present work investigates four research questions. First, can ZebraMap support a reproducible full-label-space benchmark for rare-disease diagnosis under leakage-aware splitting and ranking-based evaluation? Second, how do lexical retrieval, sparse supervised classification, dense biomedical retrieval, cross-encoder reranking, and caption-based multimodal late fusion compare within a common experimental framework? Third, can evidence-grounded explanation quality be measured quantitatively on top of frozen diagnosis outputs rather than only through qualitative examples? Fourth, how sensitive is explanation quality to the choice of open instruction model and to the introduction of support-case evidence? These questions map directly to the gap identified above: benchmark construction, model comparison, explanation assessment, and diagnosis-versus-explainability tradeoffs.

To answer these questions, ZebraMap is transformed into a downstream benchmark in which each patient case is treated as a diagnosis query and each disease profile is treated as a candidate label within a catalog of 1727 diseases. The proposed framework proceeds in five stages. Raw multimodal inputs are first normalized into case-level and disease-level benchmark artifacts. Cases are then partitioned using grouped splitting by source article to reduce article-level leakage. Each evaluation case is next processed through three text-based candidate-generation branches: BM25 lexical retrieval, a class-balanced TF–IDF classifier, and dense retrieval with MedCPT encoders. Their outputs are combined through reciprocal-rank fusion and refined through MedCPT cross-encoder reranking. Image-linked evidence is incorporated at a later stage through caption-based late fusion rather than through a learned joint text–image encoder, keeping the multimodal contribution explicit. Finally, a grounded explanation module generates matched findings, rationale statements, alternative considerations, and confirmatory-test suggestions from frozen ranking outputs. This design addresses the identified gap by supporting benchmark reproducibility, strong baseline comparison, explicit control over modality fusion, and quantitative evaluation of explanation quality without conflating diagnosis and explanation into a single model.

The contributions of this paper are as follows:

1. Benchmark formulation. ZebraMap is recast as a reproducible top-*k* ranking benchmark over 1727 rare diseases with grouped splitting by source article.
2. Hybrid diagnosis pipeline. A hybrid pipeline combining BM25, a class-balanced TF–IDF classifier, MedCPT dense retrieval, cross-encoder reranking, and caption-based late fusion attains test MRR 0.3905 and Recall@10 0.5507 on 10,895 test cases, while the class-balanced TF–IDF classifier alone reaches MRR 0.4200 and Recall@10 0.6279, so the contribution of the hybrid pipeline lies in integrating ranking with grounded explanation rather than in maximizing single-metric accuracy.
3. Grounded explanation on frozen predictions. An explanation stage operating only on frozen diagnosis outputs is evaluated on 256 cases with seven quantitative metrics, including citation coverage and faithfulness deletion; the explanation metrics are computational surrogates and not clinician-validated judgments.
4. Diagnosis–explainability tradeoff. A four-model comparison (Qwen, Mistral, Gemma, and Llama) and a support-case ablation together quantify a tradeoff between ranking accuracy and explanation richness under frozen-prediction control.

The remainder of this paper is organized as follows. Section 2 reviews the related literature on rare-disease decision support, language-model-based diagnosis, and multimodal clinical AI. Section 3 describes the benchmark construction, split protocol, diagnosis pipeline, multimodal late-fusion design, explanation framework, and evaluation metrics. Section 4 presents the benchmark results, ablations, slice analyses, and explanation-model comparison. Section 5 discusses the implications of the findings, including the role of strong sparse baselines and the observed diagnosis-versus-explainability tradeoff. Section 6 concludes the paper and outlines directions for future work.

## 2. Related Work

Prior work on rare-disease diagnosis spans clinical decision support, machine learning over long-tail disease spaces, retrieval-augmented language-model reasoning, and multimodal clinical artificial intelligence. The review is organized thematically into four parts: decision support and ranked output, language-model and retrieval-based reasoning, hybrid and multimodal approaches, and benchmark resources. This progression shows why a reproducible benchmark jointly evaluating disease ranking, multimodal evidence integration, and grounded explanation remains necessary.

### 2.1. Rare-Disease Diagnosis, Decision Support, and Ranked Output

Reviews of rare-disease decision support consistently find progress constrained by methodological fragmentation rather than by a lack of proposed methods. Faviez et al. documented substantial heterogeneity in targeted diseases, input modalities, objectives, and evaluation procedures across diagnosis-support systems [8]; scoping reviews by Schaefer et al. and Schaaf et al. reached similar conclusions regarding narrow disease coverage, inconsistent validation, and weak external testing [9,10]. These reviews clarify structural obstacles but do not define a common benchmark task or an experimental protocol for comparing diagnostic strategies under shared conditions.

A second theme concerns output structure: in rare-disease settings with underspecified early presentations, a ranked differential is more useful than a single forced label. Phenotype-driven tools formalized this early. Phenomizer ranked candidate diseases by semantic similarity between patient and disease Human Phenotype Ontology annotations [17], and LIRICAL extended the approach with a likelihood-ratio framework yielding calibrated posttest probabilities [18]. Miyachi et al. later generalized the ranked-output perspective with a learning-to-rank clinical decision-support system [7]. These systems operate on structured phenotype terms or coded symptom inventories and are not designed for free-text narratives or image-linked evidence, leaving the field without a reproducible full-label-space ranking standard over narrative-rich multimodal case reports.

### 2.2. Language Models, Retrieval Augmentation, and Rare-Disease Reasoning

Large language models (LLMs) have shifted rare-disease work toward narrative reasoning, retrieval augmentation, and prompt-based evaluation. Retrieval-augmented generation, introduced by Lewis et al. to combine parametric LLMs with non-parametric external memory [19], has since been adapted to clinical settings: Singhal et al. showed with Med-PaLM that domain-adapted LLMs reach strong multi-task medical question answering while still lagging clinicians [20]; Zelin et al. demonstrated that retrieval augmentation materially improves ChatGPT-based rare-disease diagnosis on a phenotype-oriented benchmark [11]; and Wu et al. combined dictionary-based extraction with LLMs for rare-disease phenotyping, showing the continued value of structured intermediate representations [21]. These studies suggest LLMs are most effective when supported by retrieval or structured processing rather than treated as standalone diagnostic engines.

Most LLM studies address either small-scale evaluation or phenotype extraction rather than large-scale disease ranking. RareArena substantially expands benchmark scale with screening and confirmation tasks over PubMed Central case reports, evaluated across multiple frontier LLMs [12] but remains primarily a prompting benchmark and does not compare strong lexical baselines, sparse supervised models, dense biomedical retrieval, cross-encoder reranking, and multimodal late fusion within a single downstream pipeline. The open question is therefore how LLMs should be positioned relative to retrieval, ranking, and explanation in a unified benchmark framework.

### 2.3. Hybrid and Multimodal Approaches, with Explainability Considerations

Rare-disease diagnosis should in principle benefit from multimodal reasoning because clinically relevant evidence is distributed across free text, structured findings, diagnostic procedures, and image-linked descriptions, yet multimodal gains are sensitive to fusion design and evaluation. Buess et al. surveyed generative and multimodal medical AI and argued that cross-modal integration remains limited by data-quality variation, task mismatch, and insufficiently rigorous validation [13]. This caveat is relevant here, where image-linked information is available for many but not all cases and must be incorporated in an explicit and auditable manner.

Explainability adds a further constraint: Liu et al. argued that explainable AI is clinically useful only when explanations are attached to concrete workflow needs and auditable evidence rather than weakly grounded narrative [14], and Agrawal et al. cautioned that LLM evaluation in medicine can mislead when task framing, data, and metrics are poorly aligned with clinical use [15]. Multimodality and explainability should therefore be evaluated explicitly, with ranking and explanation quality treated as related but distinct targets; no rare-disease study has yet done so within a single benchmark that preserves a clear separation between diagnosis and explanation generation.

### 2.4. Evaluation Benchmarks and Dataset Resources

Performance claims in rare-disease AI are meaningful only when task definitions, disease catalogs, and split protocols are transparent. RareArena is currently the most developed large-scale rare-disease benchmark for LLM evaluation and demonstrates the value of separating screening from confirmation [12], but its emphasis remains text-based prompting rather than a hybrid retrieval-and-ranking pipeline with multimodal late fusion and explanation analysis.

Dataset resources play a complementary but distinct role. ZebraMap consolidates disease summaries, structured case content, and linked image metadata across 1727 diseases and 69,146 structured cases [16], but a data resource does not automatically become a benchmark; it additionally requires a formal query–target structure, reproducible partitioning, baseline comparators, and explicit evaluation metrics. Table 1 summarizes representative prior works and shows that no single study jointly provides a published multimodal resource, full-label-space disease ranking, leakage-aware splitting, strong non-LLM baselines, multimodal fusion analysis, and quantitative grounded-explanation evaluation.

### 2.5. Research Gap and Positioning of the Present Study

The four themes converge on one gap. Decision-support studies justify ranked differential output but do not define a reproducible full-label-space benchmark [7,8,9,10]; LLM studies show retrieval and prompting are useful but remain limited by scale, task scope, or separation from stronger non-LLM baselines and downstream reranking [11,12,21]; multimodal and explainability studies demand rigorous evaluation but rarely situate it inside a rare-disease benchmark that separates diagnosis from explanation [13,14,15]; and published resources such as ZebraMap provide the substrate but not the protocol [16]. No prior work jointly supports full-label-space top-*k* disease ranking, leakage-aware grouped splitting, lexical and supervised baselines, biomedical dense retrieval and reranking, caption-mediated late fusion, and quantitative explanation assessment on frozen diagnosis outputs. The present work closes this gap on ZebraMap.

## 3. Materials and Methods

### 3.1. Framework Overview

**Figure 1:**
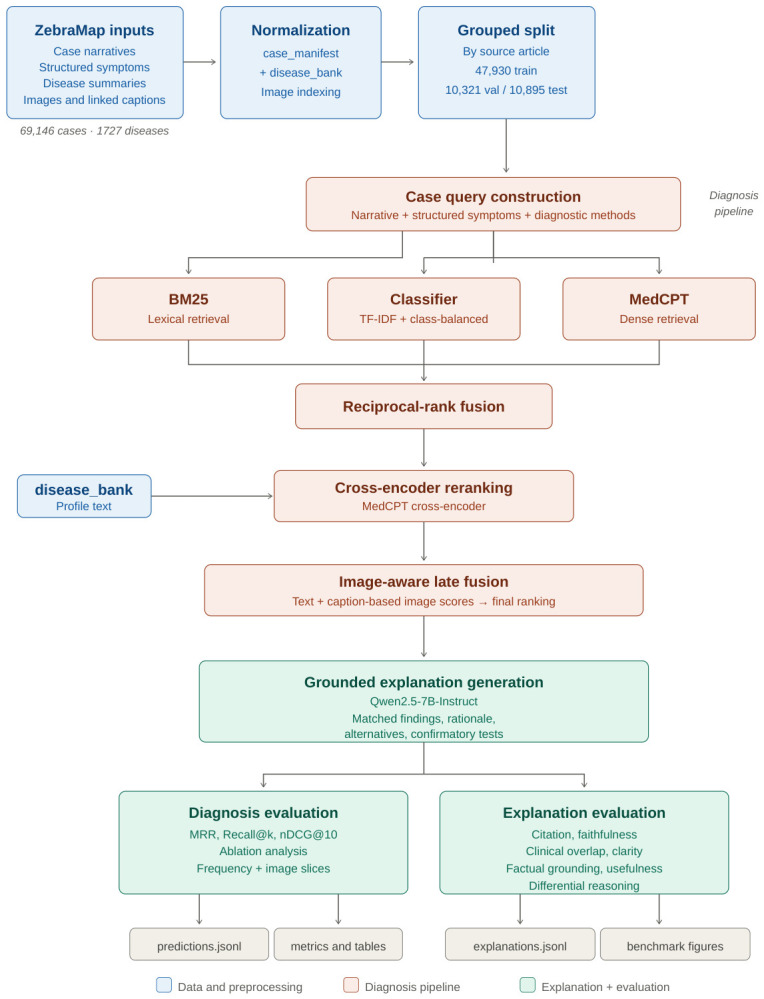
Methodological overview of the ZebraMap rare-disease diagnosis and grounded explanation framework. Raw multimodal case-report data were normalized into case-level and disease-level benchmark artifacts, followed by grouped splitting by source article to reduce leakage. Each case was then processed through lexical retrieval, supervised classification, dense retrieval, cross-encoder reranking, and caption-based image-aware late fusion before grounded explanation generation and separate diagnosis and explanation evaluation.

The benchmark framework is a sequential diagnosis-and-explanation pipeline in which disease ranking and explanation generation are treated as related but distinct stages. Figure 1 summarizes the full workflow. Raw ZebraMap resources were first normalized into two benchmark artifacts: a case-level manifest used for query construction and a disease-level bank used for retrieval, classification, and reranking. Cases were then partitioned by source article so that case reports originating from the same publication did not leak across training and evaluation splits. Each evaluation case was transformed into a unified diagnosis query and processed by three text-based candidate-generation branches: BM25 lexical retrieval, a class-balanced term frequency–inverse document frequency (TF–IDF) classifier, and dense biomedical retrieval with MedCPT. The resulting candidate lists were merged by reciprocal-rank fusion (RRF), reranked with a MedCPT cross-encoder, and rescored by caption-mediated late fusion using image-linked text. Only after the final ranked disease list was fixed was an explanation model invoked.

**Figure 2:**
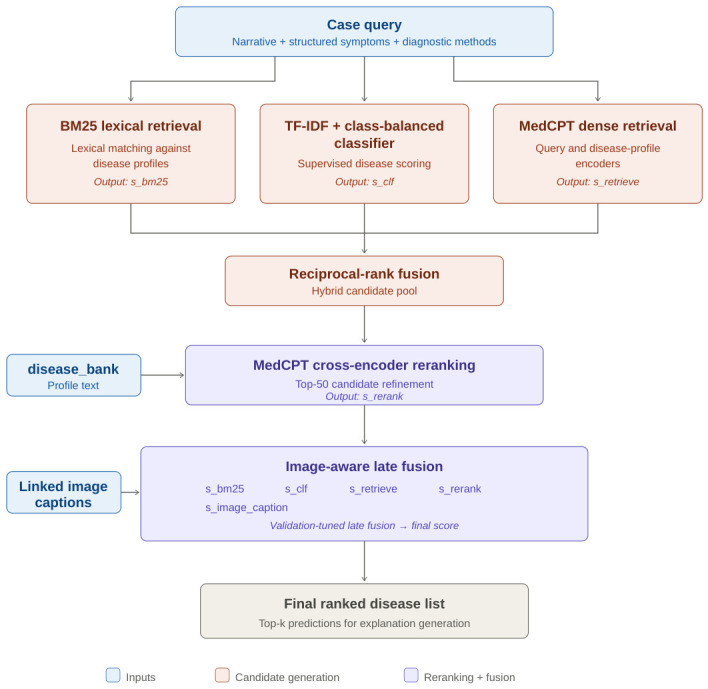
Detailed view of the hybrid diagnosis pipeline used in the frozen main benchmark run. A unified text query was processed in parallel by BM25 lexical retrieval, a class-balanced TF–IDF classifier, and MedCPT dense retrieval. The resulting candidates were merged by reciprocal-rank fusion, refined by MedCPT cross-encoder reranking, and combined with caption-based image evidence through validation-tuned late fusion to produce the final ranked disease list.

This staged design served two purposes. First, it enabled sparse, dense, and hybrid rankers to be compared within a common benchmark protocol. Second, it kept explanation generation downstream from the ranking model so that explanation quality could be studied without altering diagnosis outputs. That separation was important because one of the research questions concerned grounded explanation as an evaluable object in its own right, rather than as a by-product of a generative diagnostic model. Figure 2 expands the ranking core of the framework and shows where caption-based image evidence entered the pipeline.

### 3.2. Problem Formulation

Let $C=\{c_{1},\ldots ,c_{N}\}$ denote the set of evaluation cases and let $D=\{d_{1},\ldots ,d_{M}\}$ denote the disease catalog, where M=1727 in the full benchmark. Each case c∈C has a gold disease label y(c)∈D and is represented by a normalized query text q(c) derived from the case narrative, structured symptoms, and diagnostic-method fields. Each disease d∈D is represented by a profile text p(d) derived from disease summaries and structured disease-level fields. No case content from any split (training, validation, or test) is used to build p(d), so the disease-side representation cannot leak gold-label evidence through the case partitioning.

The ranking model assigns each pair (c,d) a score s(c,d) and returns an ordered list of diseases,

(1) $$\pi_{c}={\mathrm{argsort}}_{d\in D}\;s\left(c,d\right),$$

where higher scores indicate greater diagnostic plausibility. The rank of the true disease is denoted by

(2) $$r\left(c\right)={\mathrm{rank}}_{\pi_{c}}\left(y\left(c\right)\right).$$

A top-*k* success event occurs when the correct disease appears among the first *k* entries of $\pi_{c}$,

(3) $$z_{k}\left(c\right)=I\left[r\left(c\right)\leq k\right],$$

which is consistent with ranked-retrieval evaluation practice [6]. This formulation was selected because rare-disease decision support typically benefits from a short ordered differential rather than an immediate single-label commitment [7].

The framework separated diagnostic ranking from explanation generation. Once the final ranking had been fixed, an explanation function *g* generated a grounded explanation,

(4) $$e\left(c\right)=g\left(c,\pi_{c}^{\left(K\right)},p\left(y_{1}\right),E_{c}\right),$$

where $\pi_{c}^{\left(K\right)}$ is the top-*K* ranked list, $y_{1}$ is the top-ranked disease, and $E_{c}$ is the supporting evidence bundle assembled from case snippets, alternative predictions, and structured disease information. This decomposition made it possible to analyze diagnosis quality and explanation quality separately.

### 3.3. Candidate Generation and Hybrid Retrieval

The candidate-generation stage balances lexical precision, sparse supervised discrimination, and semantic retrieval. Each case was represented by a single query text obtained by concatenating the normalized free-text narrative with structured symptom and diagnostic-method fields. Rare-disease case reports distribute diagnostically relevant evidence across prose descriptions and structured fields; excluding either source would reduce recall in the early ranking stages. The same query representation was used across all candidate-generation branches so that performance differences reflected the retrieval or classification mechanism rather than changes in input construction.

The first branch used BM25 over disease-profile text [22]. BM25 was selected because it is a strong and interpretable probabilistic baseline for ranking tasks in which exact terminology overlap remains informative. In rare-disease case reports, diagnostically important strings such as gene names, syndromic descriptors, or radiologic findings are often repeated verbatim; a lexical branch therefore provided a useful lower-bound baseline and a complementary signal to dense retrieval. The BM25 index used the standard parameters $k_{1}=1.5$ and b=0.75, which were retained from the benchmark implementation. The lexical score for case *c* and disease *d* was

(5) $$\mathrm{BM}25\left(c,d\right)=\sum_{t\in q(c)}\mathrm{IDF}\left(t\right)\;\frac{f\left(t,p\left(d\right)\right)\left(k_{1}+1\right)}{f\left(t,p\left(d\right)\right)+k_{1}\left(1-b+b\;\frac{\left|p(d)\right|}{\bar{\left|p\right|}}\right)}.$$

The second branch used a sparse supervised classifier built from TF–IDF features [23]. TF–IDF was used because it reweights tokens by within-document prominence and corpus rarity, which is appropriate when case descriptions contain both common medical vocabulary and highly discriminative rare-disease cues. The classifier was implemented as an SGDClassifier with logistic loss, $L_{2}$ regularization, and class balancing. The classifier used unigram and bigram features, English stop-word filtering, sublinear term frequency scaling, a minimum document frequency of 2, a maximum vocabulary size of 50,000 terms, regularization strength $\alpha =10^{-5}$, a maximum of 2000 training iterations, and convergence tolerance $10^{-3}$. This branch was retained because sparse linear models remain competitive on long-tail label spaces and often outperform more complex methods when label imbalance is severe.

The third branch used dense retrieval with MedCPT query and article encoders [24]. Dense retrieval was selected to recover semantically related diseases even when case wording and disease summaries did not share exact vocabulary. The query encoder was ncbi/MedCPT-Query-Encoder, the document encoder was ncbi/MedCPT-Article-Encoder, the maximum input length was 512 tokens, and the retrieval batch size was 16. Dense similarity was computed with cosine similarity,

(6) $$s_{\mathrm{dense}}\left(c,d\right)=\cos\left(e_{q}\left(q\left(c\right)\right),e_{d}\left(p\left(d\right)\right)\right).$$

The three ranked lists were merged by RRF [25]. In the frozen main run, the hybrid retriever was enabled with per-source truncation of 100 candidates, final retrieval depth 100, candidate-pool depth 150, RRF constant K=60, and source weights $w_{\mathrm{bm}25}=1.5$, $w_{\mathrm{clf}}=0.75$, and $w_{\mathrm{dense}}=1.0$. The fusion score was

(7) $$s_{\mathrm{RRF}}\left(d\right)=\sum_{m\in M}\frac{w_{m}}{K+r_{m}\left(d\right)},$$

where $r_{m}\left(d\right)$ is the rank assigned by source *m*. The weighted RRF design was used because it preserves ranking robustness while allowing stronger signals to contribute differentially. Algorithm 1 summarizes this stage.
**Algorithm 1** Hybrid Candidate Generation and Reciprocal-Rank Fusion**Input:** case query q(c), disease catalog D, per-source depth $K_{s}$, final depth $K_{f}$.**Output:** hybrid candidate list $\pi_{c}^{\left(0\right)}$.
Score all diseases in D with BM25 over disease-profile text and retain the top $K_{s}$ results.Transform q(c) with the fitted TF–IDF vectorizer, predict class probabilities with the class-balanced linear classifier, and retain the top $K_{s}$ diseases.Encode q(c) and all disease profiles with MedCPT, compute cosine similarity, and retain the top $K_{s}$ diseases.Merge the three ranked lists with RRF using source weights {1.5,0.75,1.0} and constant K=60.Sort diseases by $s_{\mathrm{RRF}}$ and retain the top $K_{f}$ candidates.

### 3.4. Cross-Encoder Reranking and Caption-Mediated Late Fusion

The hybrid candidate list was refined in two stages. First, a cross-encoder reranker rescored each candidate using direct case–disease interaction. Second, a caption-mediated late-fusion stage added image-linked evidence without learning a shared text–image representation. This separation was deliberate: reranking was used to improve text-based ordering among plausible disease candidates, whereas the multimodal signal was introduced conservatively as an auxiliary score so that its contribution remained transparent.

Reranking used the ncbi/MedCPT-Cross-Encoder model loaded in safetensors format. For each case, the top 50 fused candidates were rescored by the cross-encoder. This design reflects the standard retrieval-then-rerank pattern in information retrieval: a high-recall candidate generator narrows the search space, and the cross-encoder then applies a more computationally expensive interaction model to the shortlist. The reranker therefore improved local ordering while preserving tractability.

Multimodal evidence was incorporated only after reranking. The benchmark did *not* learn a joint image–text embedding space and did not train an image-only classifier. Instead, image information was represented through linked caption text. A disease-specific caption bank was built from training-set image captions, and each evaluation case contributed a concatenated case-caption string when such captions were available. The auxiliary image score was computed as Jaccard-style lexical overlap between these two token sets,

(8) $$s_{\mathrm{img}}\left(c,d\right)=\frac{\left|T(g(c))\cap T(h(d))\right|}{\left|T(g(c))\cup T(h(d))\right|},$$

where g(c) is the concatenated case-caption text, h(d) is the disease caption bank, and T(·) denotes stop-word-filtered tokenization. This choice kept the multimodal contribution explicit and auditable, which was preferred over an opaque fusion module in a benchmark whose main goal was analysis rather than end-to-end model optimization.

All branch scores were min–max normalized within each case. The final late-fusion score was

(9) $$s_{\mathrm{final}}\left(c,d\right)=\alpha {\tilde{s}}_{\mathrm{bm}25}+\beta {\tilde{s}}_{\mathrm{clf}}+\gamma {\tilde{s}}_{\mathrm{ret}}+\delta {\tilde{s}}_{\mathrm{ce}}+\eta {\tilde{s}}_{\mathrm{img}},$$

where tildes denote per-case min–max normalization, $s_{\mathrm{ret}}$ is the dense retrieval score, and $s_{\mathrm{ce}}$ is the cross-encoder score. The image-rerank stage evaluated the top 20 candidates per case. Candidate weight sets were tuned on the validation split by maximizing the composite objective

(10) J=MRR+Recall@5+Recall@10+nDCG@10.

The selected frozen weights were α=0.05 for BM25, β=0.55 for the classifier, γ=0.00 for dense retrieval, δ=0.30 for reranking, and η=0.10 for the caption-based image score. We set γ=0.00 because the dense signal had already entered the pipeline upstream through reciprocal-rank fusion (Equation (7)) and the MedCPT cross-encoder reranker; adding an explicit weight on the standalone dense score did not improve the composite objective *J* on the validation split. Dense retrieval is therefore still important in candidate generation, just not in the final scoring step. Algorithm 2 summarizes this stage.
**Algorithm 2** Validation-Tuned Caption-Mediated Late Fusion**Input:** reranked candidate list $\pi_{c}^{\left(0\right)}$, case-caption text g(c), disease caption bank h(d), candidate weight set W.**Output:** final ranked list $\pi_{c}$.
For each candidate disease $d\in \pi_{c}^{\left(0\right)}$, compute the caption-overlap score $s_{\mathrm{img}}\left(c,d\right)$ using Equation (8).Min–max normalize the BM25, classifier, dense-retrieval, reranker, and image scores within case *c*.For each candidate weight vector w∈W, compute validation-set rankings with Equation (9).Select the weight vector that maximizes the objective in Equation (10) on the validation split.Apply the selected weights to the validation and test candidates and sort diseases by $s_{\mathrm{final}}\left(c,d\right)$.

### 3.5. Grounded Explanation Generation and Auxiliary Analyses

After the diagnostic ranker produced the final candidate list, the explanation stage generated a structured rationale for a fixed subset of cases. The explanation input contained five elements: the normalized diagnosis query, the top predicted disease, up to three alternative predictions, the disease-profile text, and a compact evidence bundle derived from the case narrative and retrieved support. Supporting case snippets were extracted by scoring case sentences against the top-disease profile and retaining the highest-overlap sentences. The prompt instructed the model to produce matched findings, a rationale for the leading diagnosis, a short discussion of alternatives, and suggested confirmatory tests. This format was chosen to keep explanations tied to observable evidence rather than to unconstrained free-form narrative.

In the frozen main run, explanation generation used Qwen/Qwen2.5-7B-Instruct with the transformers_generate backend, temperature 0.1, maximum generation length 384 tokens, checkpoint interval 8 cases, and maximum explanation subset size 256. The generation prompt was truncated to 2048 input tokens by the tokenizer. The explanation model did not alter the diagnosis ranking; it operated only on frozen predictions. This separation was necessary because the study was designed to compare explanation quality without introducing confounding changes in ranking accuracy.

Two auxiliary ablation families were evaluated. The first added support-case evidence during reranking and explanation. In that ablation, representative training-case snippets and a support score were appended to each candidate disease to test whether explicit case memory improved either ranking or explanation quality. The second ablation held the diagnostic predictions fixed and varied only the explanation model. Four open instruction-tuned generators were evaluated on the same 256-case subset under the same prompt template: Qwen2.5-7B-Instruct, Mistral-7B-Instruct-v0.3, Gemma-2-9B-it, and Llama-3.1-8B-Instruct. Because the predictions were frozen, any changes in this comparison reflected explanation generation alone.

### 3.6. Dataset, Preprocessing, and Benchmark Release

The experiments used the full ZebraMap release described in the original dataset paper [16]. The raw resource comprised structured case data, disease-level information, literature metadata, and a linked image directory corresponding to the full ZebraMap release. The benchmark package preserved resolved configuration, environment snapshots, intermediate candidate lists, final predictions, explanations, tables, and figures.

Preprocessing produced two benchmark artifacts. The case_manifest stored one row per case with the disease identifier, source article identifier, normalized case text, structured symptom list, diagnostic-method list, split assignment, and linked image-caption metadata. The disease_bank stored one row per disease with a consolidated profile text assembled from disease summaries and structured disease-level fields. Whitespace was normalized, lightweight stop-word filtering was applied in token-based retrieval stages, and image handling was separated from core text normalization. Cases without images were retained. Grouped splitting was performed at the source_article level to reduce article-level leakage. The disease_bank profiles use only disease-level metadata, and the disease-specific caption banks used in the late-fusion stage (Section 3.4) use only training-set image captions; no validation or test case content enters either resource. The resulting split contained 47,930 training cases, 10,321 validation cases, and 10,895 test cases, with disease counts of 1464, 1131, and 1268, respectively. The full benchmark contained 69,146 structured cases, 1727 diseases, 41,955 cases with raw images, and 51,773 cases with at least one linked indexed image, corresponding to 140,794 linked images after indexing.

The present study involved only secondary analysis of a previously published dataset derived from case reports. No new patient recruitment, intervention, or collection of identifiable private data was performed. Table 2 summarizes the benchmark release used throughout the paper.

### 3.7. Evaluation Metrics

Ranking quality was assessed with Recall@*k*, mean reciprocal rank (MRR), normalized discounted cumulative gain at *k* (nDCG@*k*), mean rank, median rank, macro recall, and weighted recall. Recall@*k* measures whether the correct disease appears within the first *k* retrieved candidates. MRR emphasizes early placement of the correct disease and is well suited to differential-diagnosis ranking because a correct disease placed at rank 1 contributes more than one placed at rank 10. nDCG@*k* was included because it rewards higher placement near the top of the list and is standard in information retrieval [6,26]. For a single relevant disease per case, the metric definitions were

(11) $$\mathrm{Recall}@k=\frac{1}{N}\sum_{i=1}^{N}I\left[r_{i}\leq k\right],$$

(12) $$\mathrm{MRR}=\frac{1}{N}\sum_{i=1}^{N}\frac{1}{r_{i}},$$

(13) $$\mathrm{nDCG}@k=\frac{1}{N}\sum_{i=1}^{N}\frac{I[r_{i}\leq k]}{\log_{2}\left(r_{i}+1\right)},$$

where $r_{i}$ is the rank of the correct disease for case *i*. Mean rank and median rank are computed only over the subset $C^{★}\subseteq \left\{1,\ldots ,N\right\}$ of test cases for which the true disease appeared in the retrieved candidate list; cases without a finite rank are not included in these two summaries. Recall@*k* is reported over the full test set. Macro recall was computed by averaging disease-level recall scores, whereas weighted recall aggregated hits over all cases.

Grounded explanation quality was evaluated with citation coverage, faithfulness deletion, clinical overlap, and four heuristic judge scores. Citation coverage measured how much explicit evidence was attached to the explanation,

(14) $$\mathrm{CC}\left(c\right)=\min\left(1,\frac{|E_{c}|}{4}\right),$$

where $E_{c}$ is the set of supporting evidence identifiers. Faithfulness deletion measured the drop in case–disease lexical overlap after removing the snippets used to support the explanation,

(15) $$\mathrm{FD}\left(c\right)=\max\left(0,J\left(x_{c},p\left(y_{1}\right)\right)-J\left(x_{c}\setminus S_{c},p\left(y_{1}\right)\right)\right),$$

where J(·,·) denotes Jaccard lexical overlap, $x_{c}$ is the case text, and $S_{c}$ is the set of supporting snippets. Clinical overlap compared matched findings in the generated explanation with structured symptoms and diagnostic-method fields,

(16) $$\mathrm{CO}\left(c\right)=\frac{|T\left(M_{c}\right)\cap T\left(U_{c}\right)|}{\left|T(U_{c})\right|},$$

where $M_{c}$ is the matched-finding text and $U_{c}$ is the structured clinical evidence. Clarity, factual grounding, differential reasoning, and usefulness were reported on a 1–5 heuristic scale computed from explanation length, number of evidence identifiers, and number of alternatives. Because no expert-annotated explanation benchmark was available for this dataset, these scores should be interpreted as operational surrogates rather than clinician-validated judgments.

### 3.8. Experimental Setup

The frozen main benchmark run was executed with random seed 42. Experiments were conducted on a server equipped with one NVIDIA H100 NVL GPU (95,830 MiB visible memory). The core software environment comprised Python 3.12.9, PyTorch 2.5.1, Transformers 4.57.6, scikit-learn 1.7.1, NumPy 2.2.2, and CUDA 12.4. Data loading and preprocessing used eight parallel workers.

All diagnosis baselines and comparison stages were evaluated under the same grouped split and the same disease catalog. The lexical baseline was BM25 [22]. The sparse supervised baseline was the class-balanced TF–IDF linear classifier built as described in Section 3.3 [23]. The dense retriever and reranker both used MedCPT [24]. The final explainable system combined these components with caption-mediated late fusion and then applied the Qwen2.5-7B explanation stage. The main benchmark was reported from one frozen seeded run. Additional experiments included one support-case ablation run and four explanation-only generator comparisons with frozen diagnosis outputs. This design isolated the effect of each component while keeping the train–validation–test partition fixed across all analyses.

Table 3 consolidates the key implementation settings needed to reproduce the frozen main run from the manuscript text alone.

### 3.9. Statistical Analysis

The study was benchmark-oriented, reporting point estimates from one frozen split and one fixed random seed. The large held-out test set (N=10,895) and consistent metric ordering across stages (Table 4) provide empirical robustness support. For the proportion metrics, Recall@*k*, Wald 95% confidence intervals and pairwise two-sample *z*-tests are reported as a footnote to Table 4. The *z*-tests assume independence between stages, which overstates the variance because the same cases are scored by every stage, so the reported *p*-values bound the paired tail probabilities from above. Bootstrap confidence intervals for MRR and nDCG@10, paired tests on per-case outcomes, and repeated runs under independent seeds require access to per-case prediction arrays that are not part of the frozen package and are therefore left to follow-up work. Systems were compared by absolute differences in ranking metrics on identical evaluation cases, by stage-wise ablation, and by subgroup analysis over disease-frequency and image-availability slices. For the explanation-model comparison, diagnosis outputs were held fixed, so differences in explanation metrics reflected only the generation stage.

## 4. Results

All results correspond to the frozen main benchmark run unless otherwise stated.

### 4.1. Main Comparison

**Figure 3:**
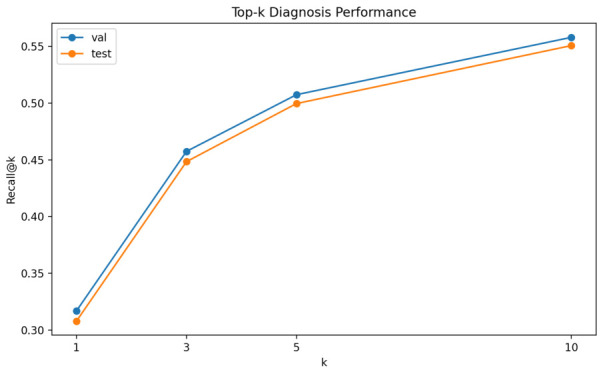
Cumulative Recall@*k* of the final explainable diagnosis pipeline on the test split of the frozen main benchmark run, plotted for k∈{1,2,…,50}.

**Figure 4:**
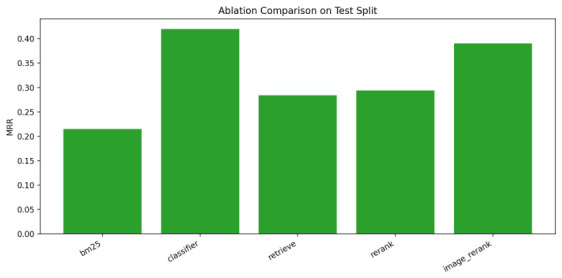
Stage-wise comparison on the frozen main benchmark run. The bars report the point estimates listed in Table 4.

The test-set comparison across diagnosis stages appears in Table 4. Among the five stages, the class-balanced TF–IDF classifier achieved the highest diagnostic accuracy (MRR 0.4200, Recall@10 0.6279), while BM25 provided the weakest baseline (MRR 0.2153). The final fusion pipeline ranked second overall (MRR 0.3905), trading peak accuracy for end-to-end diagnosis and explanation. Figure 3 shows the cumulative Recall@*k* profile of the final system, and Figure 4 visualizes the per-stage metric ordering.

Table 5 reports validation- and test-split performance of the final explainable pipeline. On the test split, the pipeline achieved MRR 0.3905 and Recall@10 0.5507. Validation-split performance was consistent (MRR 0.3987, Recall@10 0.5580), indicating that the grouped split did not introduce substantial partition bias.

### 4.2. Ablation Study

Table 6 and Table 7 report two ablation families applied after the main run. The support-case ablation introduced richer contextual evidence into both the ranking and explanation stages. Diagnosis quality declined (test MRR fell 9.2%, from 0.3905 to 0.3549; Recall@10 from 0.5507 to 0.5086), while explanation quality improved across all four metrics (Table 6), most notably citation coverage (0.7334 to 0.9961) and factual grounding (3.9336 to 4.9844).

With diagnosis rankings held fixed, only explanation quality varied across generators (Table 7). Qwen2.5-7B-Instruct produced the strongest grounding profile, while Llama-3.1-8B-Instruct yielded the highest clarity score (4.0193). Mistral-7B-Instruct-v0.3 and Gemma-2-9B-it performed comparably on all metrics.

### 4.3. Sensitivity Analysis

**Figure 5:**
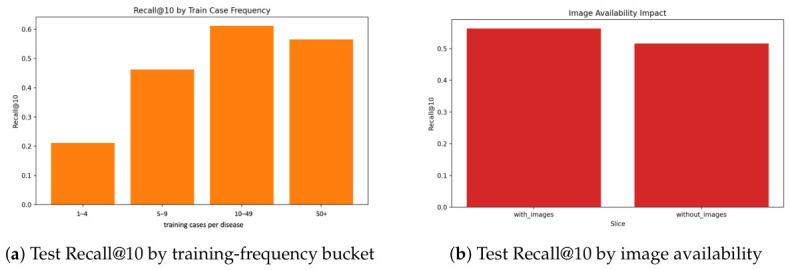
Sensitivity analysis for the frozen main benchmark run. The left panel shows Recall@10 across disease-frequency buckets, and the right panel shows Recall@10 by image availability.

Table 8 and Figure 5 report sensitivity across disease-frequency and image-availability strata. Performance increased monotonically across training-frequency buckets (Table 8), ranging from MRR 0.0936 for the rarest diseases (1–4 training cases) to MRR 0.4466 for diseases with 10–49 examples. The drop at the 50+ bucket (MRR 0.4048) suggests diminishing returns at higher frequency, possibly reflecting greater phenotypic variability within more prevalent disease categories.

Cases with linked image captions outperformed those without (MRR 0.4019 vs. 0.3579; Recall@10 0.5629 vs. 0.5161; Table 8), confirming that caption-mediated late fusion contributed positively when image-linked evidence was available. Figure 5 visualizes the Recall@10 values for both slice types.

### 4.4. Qualitative Results

**Figure 6:**
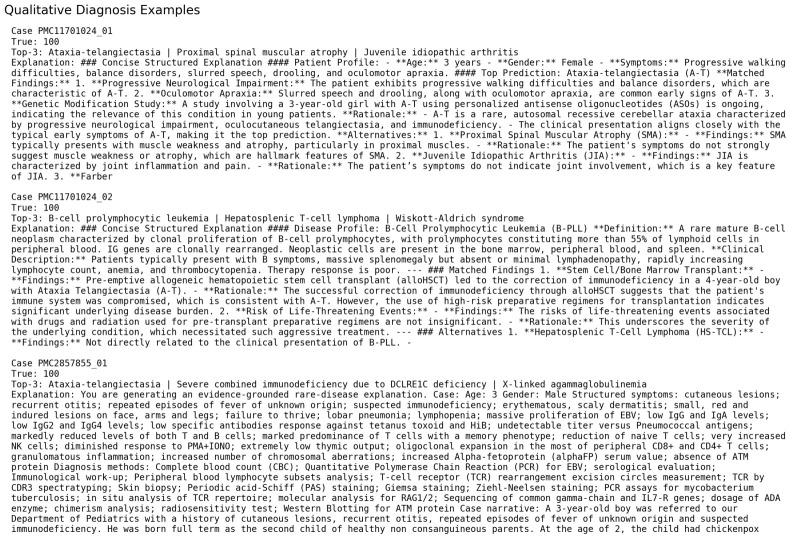
Three representative cases from the frozen main benchmark run: a top-1 correct example for *Ataxia-telangiectasia*, a case where the correct disease appeared in the top-*k* list but not at rank 1, and a failure case with biologically plausible alternatives.

Figure 6 presents three representative cases from the frozen main benchmark run: a top-1 correct example, a case in which the correct diagnosis appeared within the top-*k* list but not at rank 1, and a failure case with clinically plausible alternatives. In the top-1 correct example, a pediatric presentation with progressive gait difficulty, balance impairment, slurred speech, drooling, and oculomotor apraxia was ranked as *Ataxia-telangiectasia* at rank 1. In the second case, the correct disease was retained within the top-ranked list while an alternative diagnosis occupied rank 1. In the failure case, high-salience immune-system findings redirected the ranking toward a hematologic condition, although the explanation preserved evidence-linked alternatives in the output.

### 4.5. Computational Efficiency

Table 9 summarizes the recorded runtime characteristics of the frozen sequential run. The benchmark package preserved stage-completion markers and a device snapshot, but it did not preserve per-model parameter counts or FLOP counters. The recorded device snapshot showed one NVIDIA H100 NVL GPU with 95,830 MiB of visible memory. From the completion markers, the interval between the end of preparation and the final report was 10 h 45 min 48 s. The longest recorded interval occurred between reranking and completion of image-aware late fusion, which was 5 h 13 min 02 s. This duration reflects the sequential processing of caption-overlap scores for all 21,216 evaluation cases against a 1727-disease caption bank; a parallelized implementation would substantially reduce this stage. The explanation stage completed 256 cases in 33 min 08 s.

### 4.6. Statistical Significance

Wald 95% confidence intervals and pairwise two-sample *z*-tests for Recall@*k* are reported in the footnote to Table 4. The stage-wise differences in Recall@10 are roughly seven to thirty times the corresponding Wald half-width (±0.009), and the *z*-statistics for every pair involving the final fusion or classifier rows exceed 9.5 (p<0.001 even under the conservative independence assumption). Bootstrap intervals for MRR and nDCG@10 and paired-test variants are left to follow-up work (see Section 3.9).

## 5. Discussion

### 5.1. Diagnostic Performance and Task Difficulty

On the test split, the final fusion pipeline (MRR 0.3905) ranked second behind the class-balanced TF–IDF classifier (MRR 0.4200), reflecting the design choice to integrate ranking with grounded explanation rather than to maximize accuracy on a single metric. The results show that the proposed hybrid pipeline produces ranked differentials across a large and imbalanced rare-disease space. The final explainable pipeline achieved test MRR 0.3905, Recall@1 0.3078, and Recall@10 0.5507, placing the correct disease within the top 10 candidates in more than half of all test cases. The gap between Recall@1 and Recall@10 shows that a substantial fraction of cases contained enough signal for the correct diagnosis to appear near the top of the ranked list even when not placed first. That pattern is consistent with the clinical goal of ranked differential diagnosis, which aims to surface the correct disease within a manageable review list rather than force a single-label commitment [7].

The slice analysis clarifies where performance is lowest. Diseases with only 1–4 training cases reached test MRR 0.0936 and Recall@10 0.2115, whereas the 10–49 bucket reached MRR 0.4466 and Recall@10 0.6115. This contrast indicates that data sparsity in the long tail, rather than model architecture alone, is the primary limiting factor for rare-disease diagnosis in this setting. Clinical deployment would therefore require strategies targeting ultra-rare conditions, such as few-shot learning, external knowledge bases, or specialist-curated disease profiles [8].

### 5.2. Pipeline Component Analysis and the Role of Sparse Supervision

The stage-wise comparison clarifies which components contributed most to ranking quality. BM25 provided an interpretable lexical baseline (test MRR 0.2153, Recall@10 0.3441). Dense retrieval and cross-encoder reranking improved those values to MRR 0.2842 and 0.2937, respectively, indicating that semantic matching and pairwise interaction modeling were both useful in this disease space. However, the strongest single stage remained the class-balanced TF–IDF classifier, establishing a sparse baseline that future methods must exceed under the same split protocol.

The explainable pipeline (MRR 0.3905) fell below the classifier baseline (MRR 0.4200), reflecting a deliberate design tradeoff: separating diagnosis and explanation preserves interpretability but relinquishes the accuracy gains of a single optimized ranking stage. Future work may pursue joint optimization of ranking and explanation within a single model while maintaining this interpretive separation. The selected late-fusion weights assigned the largest coefficient to the classifier signal (β=0.55), a smaller weight to reranking (δ=0.30), and a still smaller weight to caption-mediated image evidence (η=0.10). This weighting pattern is consistent with the observed accuracy profile: in the present configuration, the most reliable signal remained supervised sparse text matching, while dense retrieval, reranking, and image-linked evidence contributed complementary but weaker gains. Although γ=0.00 zeroes the dense term in the final scoring step, dense retrieval still contributes upstream through reciprocal-rank fusion in the candidate generator and through the cross-encoder reranker; the hybrid design therefore operates as a staged combination across candidate generation and reranking rather than as a balanced linear blend in the final score.

The multimodal component should be interpreted precisely. Image information entered the pipeline only through caption-based late fusion rather than through a learned shared image–text encoder. Cases with linked images achieved test MRR 0.4019 and Recall@10 0.5629, compared with MRR 0.3579 and Recall@10 0.5161 for cases without linked images. These numbers indicate that image-linked information was associated with better performance in the present pipeline, but they do not indicate that the full visual content of ZebraMap was modeled.

### 5.3. Grounded Explanation and Diagnosis–Explainability Tradeoffs

The explanation results add a second dimension to the benchmark. On the fixed 256-case subset, the main explanation model achieved citation coverage 0.7334, clinical overlap 0.4609, factual grounding 3.9336, and usefulness 3.8734. These values do not constitute clinician validation, but they show that explanation quality can be quantified systematically rather than reported only through selected examples. The open-model comparison showed that Qwen2.5-7B-Instruct produced the strongest grounding profile among the tested generators, whereas Llama-3.1-8B-Instruct yielded the highest clarity score.

The support-case ablation exposed a more consequential finding. When representative training-case evidence was introduced, explanation quality improved across citation coverage, clinical overlap, factual grounding, and usefulness, while diagnosis quality fell (test MRR from 0.3905 to 0.3549; Recall@10 from 0.5507 to 0.5086). Support-case evidence therefore altered ranking quality and explanation quality in opposite directions. This pattern suggests that richer contextual evidence can improve explanation grounding without necessarily improving the ranking function that produced the explanation.

One possible reason for this divergence is that support cases bring in vocabulary from training neighbours that are lexically similar but diagnostically heterogeneous. The added vocabulary is useful for the explanation stage because the generator has more concrete features to cite, but it dilutes the classifier signal in the ranking stage, since competing diseases gain surface-token overlap. Routing support evidence into the explanation context only, without injecting it into the ranking features, would test this. The interpretation remains a hypothesis pending controlled ablations.

### 5.4. Limitations and Implications

Several aspects of the design bound the claims of this work and define its scope. The benchmark is built from published case reports, so it inherits the biases and reporting granularity of that literature. The explanation metrics are computational surrogates, and a prospective clinician-validation study with collaborating specialists is planned as the next step. For Recall@*k* we report Wald 95% confidence intervals and pairwise *z*-tests in the footnote to Table 4; bootstrap intervals for MRR and nDCG@10, paired tests on per-case outcomes, and repeated seeded runs are left to follow-up work since they require access to per-case prediction arrays that are not part of the frozen package. The multimodal component uses caption-mediated late fusion rather than a learned vision–text encoder such as BiomedCLIP, which is the main direction for the next iteration. Evaluation is also limited to ZebraMap, and cross-benchmark transfer to RareArena [12], Orphanet-derived, and HPO-annotated corpora is deferred because the task formulations differ.

Despite these constraints, the work makes a concrete contribution to rare-disease diagnosis research. By combining lexical, supervised, and dense retrieval components with caption-mediated multimodal fusion and grounded explanation, hybrid AI pipelines are shown to produce ranked differentials covering more than half of correct diagnoses within the top 10 candidates across 1727 diseases. The resulting evaluation framework, with explicit splits, reproducible artifacts, strong baselines, and grounded-explanation analysis, provides a foundation for improving computational support for the rare-disease diagnostic process.

## 6. Conclusions

Rare-disease diagnosis remains difficult because the evidence available at first presentation is incomplete, heterogeneous, and distributed across a long-tail disease space of thousands of conditions. This work developed and evaluated a hybrid multimodal pipeline that combines grouped article-level splitting, text-first query construction, BM25 lexical retrieval, a class-balanced TF–IDF classifier, MedCPT dense retrieval and cross-encoder reranking, caption-mediated late fusion, and a downstream grounded explanation stage operating on frozen diagnosis outputs.

Applied to 1727 rare diseases and 10,895 test cases, the final explainable pipeline achieved MRR 0.3905 (Recall@10 0.5507), placing the correct diagnosis within the top 10 candidates in more than half of cases. The class-balanced TF–IDF classifier established a supervised baseline at MRR 0.4200 (Recall@10 0.6279). The support-case variant improved citation coverage to 0.9961 and factual grounding to 4.9844 at the cost of reduced ranking quality (MRR 0.3549), confirming a diagnosis–explainability tradeoff that future work should resolve. Among tested explanation generators, Qwen2.5-7B-Instruct produced the strongest grounding profile.

These results show that a hybrid retrieval-and-classification approach, extended with caption-mediated multimodal evidence and grounded explanation, can support rare-disease differential diagnosis at scale. The framework supports direct comparison among lexical, supervised, dense, reranking, multimodal, and explanation-oriented components under one leakage-aware protocol; the pipeline outputs are intended to assist specialist review through ranked candidate differentials and grounded explanations rather than to drive autonomous clinical decision-making. Three directions follow from the reported findings: redesigning support-case integration so that richer evidence improves rather than reduces ranking quality, fine-tuning biomedical retrievers and rerankers on this disease space, and replacing heuristic explanation scores with clinician-annotated assessment to bring the pipeline closer to clinical relevance. Cross-benchmark evaluation on adapted versions of RareArena, Orphanet-derived, and HPO-annotated corpora, together with the addition of a learned vision–text encoder, are further directions for future work.

## Figures and Tables

**Table 1:** Comparative summary of representative prior works. Metrics are reproduced as reported by the cited sources and are not normalized across datasets, disease catalogs, or task formulations.

Reference	Year	Approach/Method	Dataset(s)	Key Idea	Strengths	Limitations
Miyachi et al. [7]	2023	Learning-to-rank clinical decision-support system	Validated clinical cases; not rare-disease-specific	Uses ranked candidate lists to support physician review	Formalizes ranking as a clinically relevant diagnostic output	Not a large-scale rare-disease benchmark
Zelin et al. (RareDxGPT) [11]	2024	Retrieval-augmented ChatGPT-based rare-disease diagnosis	30 cases/30 diseases; external corpus of 717 diseases	Shows that retrieval improves LLM diagnosis on a small benchmark	Shows value of retrieval augmentation	Small-scale; text-only; limited disease coverage
Wu et al. [21]	2024	Hybrid dictionary + LLM framework for rare-disease phenotyping	Unstructured clinical reports	Combines structured extraction and generative reasoning	Shows value of hybrid processing and intermediate structure	Focuses on phenotyping, not full disease ranking
Chen et al. (RareArena) [12]	2026	Large-scale LLM rare-disease benchmark	Screening: 49,760/4597; confirmation: 22,901/3522	Scales text-based rare-disease benchmarking for multiple frontier LLMs	Strong benchmark scale and systematic LLM comparison	Primarily a prompting benchmark; no hybrid retrieval-reranking-explanation pipeline
Islam et al. (ZebraMap) [16]	2025	Multimodal rare-disease dataset construction	69,146 structured cases/1727 diseases	Builds a case-linked multimodal rare-disease resource	Provides a substrate for downstream diagnosis research	Resource contribution rather than a benchmark-and-analysis framework
This work	2026	Hybrid multimodal pipeline for rare-disease diagnosis and grounded explanation	69,146 structured cases/1727 diseases	Combines leakage-aware top-*k* ranking with quantitative explanation evaluation for rare-disease differential diagnosis	Integrates ranking, multimodal late fusion, and grounded-explanation analysis	Final explainable pipeline remains slightly below the strongest classifier-only baseline

**Table 2:** Dataset and split summary for the frozen main benchmark package.

Statistic	Value
Diseases in benchmark catalog	1727
Structured case reports	69,146
Cases with raw images in source release	41,955
Cases with linked images after indexing	51,773
Total linked images	140,794
Training cases	47,930
Validation cases	10,321
Test cases	10,895
Evaluation prediction rows (validation + test)	21,216
Explanation subset size	256

**Table 3:** Key implementation settings for the frozen main benchmark run.

Component	Configuration
Random seed	42
Runtime device	CUDA; 1 × NVIDIA H100 NVL GPU with 95,830 MiB visible memory
Software versions	Python 3.12.9; PyTorch 2.5.1; Transformers 4.57.6; scikit-learn 1.7.1; NumPy 2.2.2; CUDA 12.4
Workers	8
BM25	$k_{1}=1.5$, b=0.75
TF–IDF vectorizer	word 1–2 g; English stop-word filtering; min_df = 2; max_features = 50,000; sublinear term frequency
Classifier	Stochastic gradient descent with logistic loss; $L_{2}$ regularization; $\alpha =10^{-5}$; maximum 2000 iterations; convergence tolerance $10^{-3}$; class-balanced weighting; seed 42
Dense retrieval	MedCPT bi-encoder; top-*k* = 100; candidate-pool top-*k* = 150; batch size 16; max length 512
Hybrid retrieval	per-source top-*k* = 100; RRF constant 60; source weights (1.5,0.75,1.0) for BM25, classifier, dense retrieval
Cross-encoder reranking	MedCPT cross-encoder; rerank top-*k* = 50; use_safetensors = true
Late fusion	caption-overlap backend; tune weights on validation; final selected weights (0.05,0.55,0.00,0.30,0.10) for BM25, classifier, dense retrieval, cross-encoder, image caption score
Explanation generation	Qwen2.5-7B-Instruct; max_cases = 256; max_new_tokens = 384; temperature 0.1; checkpoint interval 8

**Table 4:** Test-set comparison of the main diagnosis stages on the frozen grouped split. Values are point estimates over the held-out cases from one frozen run. The best value in each column is shown in bold and the second-best value is underlined. Wald 95% confidence intervals and pairwise *z*-tests for Recall@*k* are summarized in the table note below.

Stage	MRR	R@1	R@10 ^†^	nDCG@10
BM25	0.2153	0.1501	0.3441	0.2395
Classifier	**0.4200**	**0.3100**	**0.6279**	**0.4634**
Dense retrieval	0.2842	0.1883	0.4757	0.3213
Cross-encoder reranking	0.2937	0.2066	0.4848	0.3310
Final fusion	0.3905	0.3078	0.5507	0.4273

^†^ Recall@*k* is a binomial proportion over n=10,895 test cases. Wald 95% confidence intervals using $\hat{p}\pm 1.96\sqrt{\hat{p}\left(1-\hat{p}\right)/n}$ give half-widths of approximately ±0.009 for Recall@10 (for example, classifier [0.619,0.637]; final fusion [0.541,0.560]). The Recall@10 gap between the classifier and the final fusion (0.077) corresponds to z=11.6 under a two-sample *z*-test of proportions (p<0.001); the remaining stage pairs that involve the classifier or final-fusion rows are similarly significant. The independence assumption is conservative because the same cases are scored by every stage, so paired tests on per-case binary outcomes would yield tighter intervals. Bootstrap confidence intervals for MRR and nDCG@10 are left to follow-up work.

**Table 5:** Validation- and test-split performance of the final explainable diagnosis pipeline. Median rank and mean rank are computed over the subset of cases for which the true disease appeared in the retrieved candidate list (see Section 3.7); Recall@*k*, MRR, and nDCG@10 are computed over the full split.

Split	MRR	R@1	R@3	R@5	R@10	nDCG@10	Median Rank	Mean Rank
Validation	0.3987	0.3168	0.4574	0.5074	0.5580	0.4354	1.0	2.95
Test	0.3905	0.3078	0.4484	0.4996	0.5507	0.4273	1.0	3.00

**Table 6:** Support-case ablation. The first row corresponds to the frozen main pipeline, and the second row corresponds to the support-case variant. Diagnosis metrics are reported on the test split; explanation metrics are reported on the fixed 256-case explanation subset.

System	MRR	R@1	R@10	Citation Cov.	Clinical Ovlp.	Factual Grnd.	Useful
Main pipeline	0.3905	0.3078	0.5507	0.7334	0.4609	3.9336	3.8734
Support-case variant	0.3549	0.2818	0.5086	0.9961	0.5045	4.9844	4.2322

**Table 7:** Open-model comparison for explanation generation on the same fixed 256-case subset. Diagnosis metrics were unchanged across rows because the prediction file from the main run was held constant.

Model	Citation Cov.	Clinical Ovlp.	Factual Grnd.	Clarity	Useful	MRR/R@10
Qwen2.5-7B-Instruct	0.7334	0.4609	3.9336	3.6867	3.8734	0.3905/0.5507
Mistral-7B-Instruct-v0.3	0.4961	0.3637	2.9844	3.4083	3.4642	0.3905/0.5507
Gemma-2-9B-it	0.4961	0.3637	2.9844	3.8077	3.5974	0.3905/0.5507
Llama-3.1-8B-Instruct	0.4961	0.3637	2.9844	4.0193	3.6679	0.3905/0.5507

**Table 8:** Selected sensitivity results on the test split of the frozen main benchmark run.

Slice Type	Slice Value	Cases	MRR	R@1	R@10
Training frequency	1–4	624	0.0936	0.0385	0.2115
Training frequency	5–9	417	0.2614	0.1727	0.4628
Training frequency	10–49	2337	0.4466	0.3586	0.6115
Training frequency	50+	7517	0.4048	0.3219	0.5649
Image availability	with images	8062	0.4019	0.3182	0.5629
Image availability	without images	2833	0.3579	0.2785	0.5161

**Table 9:** Recorded runtime characteristics of the frozen sequential run. Intervals were computed from consecutive stage-completion markers; the package did not preserve the start time of the initial preparation stage, model parameter counts, or FLOP counters.

Stage	Scope	Recorded Interval
Index images	69,146 cases; 51,773 linked-image cases	00:42:39
Split	69,146 cases	01:18:02
BM25	21,216 validation + test cases	00:13:54
Classifier	train + validation + test processing	00:20:12
Dense retrieval	21,216 validation + test cases	00:07:06
Cross-encoder reranking	21,216 validation + test cases	01:46:20
Image-aware late fusion	21,216 validation + test cases	05:13:02
Explanation generation	256 cases	00:33:08
Report generation	21,216 predictions; 256 explanations	00:31:24

## Data Availability

The ZebraMap dataset analyzed in this study is publicly described in Reference [16] and is openly available on Zenodo at https://doi.org/10.5281/zenodo.17623607, accessed on 17 May 2026 under the Creative Commons Attribution 4.0 International License (CC BY 4.0).

## Abbreviations

The following abbreviations are used in this manuscript:

BM25	Best Matching 25
CDS	Clinical decision support
LLM	Large language model
MedCPT	Medical Contrastive Pre-trained Transformers
MRR	Mean reciprocal rank
nDCG	Normalized discounted cumulative gain
RRF	Reciprocal-rank fusion
TF–IDF	Term frequency–inverse document frequency
